# Effect of Sublethal Concentrations of Zinc Oxide Nanoparticles on *Bacillus cereus*

**DOI:** 10.3390/pathogens12030485

**Published:** 2023-03-19

**Authors:** Anna Krzepiłko, Katarzyna Magdalena Matyszczuk, Agata Święciło

**Affiliations:** 1 Department of Biotechnology, Microbiology and Human Nutrition, University of Life Sciences in Lublin, 20-950 Lublin, Poland; 2 Department of Environmental Microbiology, Faculty of Agrobioengineering, University of Life Sciences in Lublin, 20-069 Lublin, Poland

**Keywords:** *Bacillus cereus*, ZnO nanoparticles, toxicity, oxidative stress, biofilm, sporulation, Evans blue decolouration

## Abstract

Zinc oxide nanoparticles (ZnONPs), which are produced on a large scale, pose a potential threat to various environments because they can interact with the microbial populations found in them. Bacteria that are widespread in soil, water, and plant material include the *Bacillus cereus* group, which plays an important role in biodegradation and the nutrient cycle and is a major factor determining ecological balance. This group includes, among others, the foodborne pathogen *B. cereus sensu stricto* (herein referred to as *B. cereus*). The aim of this study was a comprehensive assessment of the effects of commercially available ZnONPs on *B. cereus*. The MIC (minimum inhibitory concentration) for *B. cereus* was 1.6 mg/mL, and the MBC (minimum bactericidal concentration) was 1.8 mg/mL. Growth of *B. cereus* was inhibited by a concentration of ZnONPs lower than or equal to MIC50. Concentrations from 0.2 to 0.8 mg/mL inhibited the growth of these bacteria in liquid media, induced symptoms of oxidative stress, and stimulated an environmental stress response in the form of biofilm and endospore formation. In addition, ZnONPs negatively affected the ability of the bacteria to break down the azo dye Evans Blue but enhanced the antimicrobial properties of phenolic compounds. Sublethal concentrations of ZnONPs generally decreased the activity of *B. cereus* cells, especially in the presence of phenolics, which indicates their potential toxicological impact, but at the same time they induced universal defence responses in these cells, which in the case of potential pathogens can hinder their removal.

## 1. Introduction

*B. cereus sensu stricto* (hereafter: *B. cereus*) is a Gram-positive aerobic sporulating bacillus [1]. It belongs to the *Bacillus cereus* group, comprising more than 50 species, of which many are phylogenetically closely related. It is capable of growth in an environment with minimal water activity of 0.93 and a pH of 4.6 and 9.5, minimum and maximum, respectively. The cardinal temperatures for the growth of these bacteria are minimum 4 °C, optimum 37 °C, and maximum 55 °C [2]. *B. cereus* bacteria are widespread in a variety of environments. They are found in decomposing organic matter, soil, water, and the gastrointestinal tracts of invertebrates, and are also a component of the microbiota of raw milk [3,4]. Bacteria of this group associated with the soil environment play an important role in the biodegradation process of natural and synthetic compounds and the nutrient cycle, and are a major factor determining ecological balance [5,6]. *B. cereus* is also counted among food pathogens. It causes outbreaks of food poisoning with vomiting or diarrhoea [7]. Two characteristics of *B. cereus*, spore formation and biofilm-forming ability, make it resistant to various environmental stressors and allow it to colonize a variety of environments. Because of these traits, these bacteria pose a major threat to food safety and human health, as they are difficult to remove using standard methods and techniques [8]. The vegetative forms and spores of these bacteria, whose source is soil, often contaminate food products of plant origin [9]. Heat-resistant spores have been detected in raw and cooked food [10]. *B. cereus* has also been isolated from meat and milk products, rice, spices, and flour samples [11,12,13,14]. Due to its psychrotrophic properties, it is able to multiply at refrigeration temperature and thereby reduce the shelf-life of ready-to-eat food, especially milk and dairy products [4,10]. It is the most common cause of spoilage of dairy products, even those that have undergone sterilization [9,15]. Due to their common presence in the environment, spores of *B. cereus* have also been detected in many raw materials used in body care products [2,16]. *B. cereus* was isolated from contaminated cosmetics, possibly due to the use of ingredients that promote the development of these bacteria, as well as to unsanitary habits of consumers [17]. This type of pollution should be controlled also in the hospital environment, because the bacterium can cause localized infection of post-surgical or trauma wounds and is a rare but significant pathogen of the eye, where it may result in severe endophthalmitis often leading to loss of vision. *B. cereus* keratitis (inflammation of the cornea) has been associated with the use of contact lenses [2]. In the scientific literature, there are also reports describing cases of skin infections caused by *B. cereus*, both in immunocompromised people and in completely healthy people without a history of injury [18,19].

However, it should be emphasized that *B. cereus* is mainly the cause of food poisoning and diarrhoea in humans [4,8]. The pathogenicity of bacteria of the *B. cereus* group is linked to their ability to produce toxins and enzymes inducing diarrhoea and vomiting in humans: phospholipase C, emetic toxin, enterotoxin complexes HBL (Hemolysin BL), NHE (Non-haemolytic enterotoxin), haemolysins (I–IV), enterotoxins (T and FM), and sphingomyelinase [20]. Emetic strains of *B. cereus* can secrete a highly toxic and heat-stable peptide into food, where it can withstand cooking temperature and induce vomiting after ingestion [21]. *B. cereus* has been isolated from biofilms found in production lines of various food processing plants, such as those processing poultry, dairy products, meat, and vegetables. In food processing, these bacteria are a potential source of recurrent cross-contamination and contamination of finished products. They can cause food spoilage or disease following consumption of contaminated food [22]. *B. cereus* cells in a biofilm are more resistant to chemical disinfectants than planktonic forms [23]. Various means of eliminating *B. cereus* are described, using detergents, ultrasound [24], essential oils [25], and combinations of stress conditions (temperature, pH, water activity (Aw), and ethanol) [26]. 

Metal nanoparticles are increasingly used as antibacterial agents. Engineered zinc oxide nanoparticles (ZnONPs), widely used in in various sectors such as medical, food processing, fuel engineering, agricultural, textile industries or detergents, and cosmetic products have a particularly high potential. This nanomaterial is also an ingredient in many medicines, especially dermatological medications and body care products. ZnO nanoparticles are used as an inorganic physical sunscreen, because they are effective at reflecting and scattering UVA and UVB radiation while preventing skin irritation, and products containing them are pleasant to the touch [27]. Zinc oxide nanoparticles are widely used in cosmetic products, e.g., toothpaste, mouthwash, and sun creams [28]. Zinc oxide is generally recognized as safe (GRAS) by the FDA (Food and Drug Administration); it induces toxic effects at much higher concentrations than those that kill microbes [29]. ZnO nanoparticles have antimicrobial properties against many pathogenic bacteria posing a potential threat to people, such as *E. coli*, *S. aureus*, *P. aeruginosa*, *Sarcina lutea*, *Klebsiella pneumoniae*, and *Proteus vulgaris*, as well as against yeasts and moulds: *S. cerevisiae*, *Candida albicans*, *A. alternata*, *R. stolonifer B. cinerea*, and *Aspergillus niger* [30,31,32,33,34,35,36]. The antimicrobial properties of zinc oxide limit the development of pathogens in products used in agriculture, the food industry, health protection, and cosmetics. 

Many works define the toxicity of ZnONPs for microorganisms by determining the MIC, MBC, or size of growth inhibition zones. Many of these experiments use biologically synthesized zinc oxide nanoparticles with high antimicrobial activity. However, nanoparticles synthesized by chemical methods are used on a large scale in industry, and it is these that can contaminate the environment. 

The present study is an attempt to explain cellular and molecular mechanisms associated with the biological activity of commercially produced ZnONPs. The effects of these nanoparticles were studied on *B. cereus* cells. *B. cereus* is a model organism for soil microorganisms, as well as for opportunistic human pathogens of importance for public health. To better understand the response of *B. cereus* to the presence of commercial ZnONPs, we investigated the response of these bacteria to concentrations of ZnONPs under 50% of the MIC. The criteria adopted for ZnONP toxicity were the capacity of the bacterial cells for planktonic growth, free radical production, spore formation, biofilm-forming capacity, and biotransformation of complex compounds (the azo dye Evans Blue). The possibility of a synergistic effect of ZnO nanoparticles and selected phenolic compounds on *B. cereus* was tested as well, to simulate the environment of cosmetic products contaminated with these bacteria, as such products often contain plant extracts or their components in addition to ZnONPs.

## 2. Materials and Methods

Commercially produced zinc oxide nanoparticles (ZnONPs) were obtained from Sigma Aldrich (catalogue no. 721077). 

### 2.1. Bacterial Strain

*Bacillus cereus* strain PCM 1948, 2019 was purchased from the Polish Collection of Microorganisms and stored at −80 °C in 15% glycerol solution. Immediately before the experiments, the strain was thawed and inoculated into nutrient broth. The culture was conducted at 37 °C for 24 h. 

### 2.2. Determination of Growth Parameters

The microdilution technique in liquid broth was used to determine the MIC, i.e., the minimum concentration of an antimicrobial agent (in mg/mL) that inhibits the growth of microorganisms, measured as OD [37]. A series of dilutions of ZnONPs (from 1.4 μg/mL to 2 mg/mL) was prepared in medium and inoculated with a bacterial inoculum so that the final concentration was 5 × 10^4^ CFU/mL. The solutions were placed in a BioScreen 100-well plate and incubated at 37 °C with shaking for 48 h. Optical density was measured at 600 nm using a Bioscreen C reader. The cultures from the wells in which the OD value did not change over time during incubation were collected and plated on solid nutrient agar. A bacterial count of 6.5 × 10^8^ CFU/mL was obtained in the control sample. The MIC value was read for the lowest concentrations at which there was no bacterial growth. The MBC value was determined as the lowest concentration of ZnONPs expressed in mg/mL at which 99.9% of bacteria die [37]. 

### 2.3. Bacterial Cultures in Liquid Medium with ZnONPs at Concentrations below the MIC

To assess the response of bacteria to concentrations of nanoparticles below the MIC, a liquid nutrient broth medium containing various concentrations of ZnONPs (0.2 mg/mL; 0.4 mg/mL; 0.6 mg/mL; 0.8 mg/mL) was prepared. The medium was inoculated with a 24 h inoculum of bacteria, so that the final concentration of bacterial cells in the medium was 5 × 10^4^ CFU/mL, and incubated at 37 °C. After 48 h of culture the OD was measured, and NBT, endospore formation, and biofilm formation tests were performed. In each test, a sample without ZnONPs was used as a negative control.

### 2.4. Evaluation of Sporulation Rate

A fixed microscope slide was prepared from the cultures growing in the presence of various concentrations of ZnONPs for 48 h. Then the slide was stained by the Schaeffer–Fulton method using malachite green and safranin [38]. An optical microscope was used to count all the cells and endospore-forming cells in 20 fields of view. The number of spores in the slides was expressed as a percentage.

### 2.5. Determination of Superoxide Radicals

In the *B. cereus* cultures incubated with ZnONPs, the concentration of superoxide radicals was determined by spectrophotometry using nitro blue tetrazolium (NBT). In an alkaline environment, superoxide anions cause NBT to form formazan [39]. The absorbance of the formazan produced is proportional to the amount of anion radicals generated and is a measure of oxidative stress. A reaction mixture containing 0.2 mL *B. cereus* culture, 0.05 mL 1 M NaOH, 0.1 mL 5 mM NBT solution, and 2.65 mL distilled water was used as a control. The test samples additionally contained ZnONPS at concentrations of 0.2, 0.4, 0.6, and 0.8 mg/mL. Due to the possibility that ZnONPs alone could generate superoxide anion radicals in the reaction environment, a negative control without bacterial cells was used. All samples were incubated in the dark for 30 min at 20 °C, and then the absorbance was measured at 560 nm. The level of superoxide anion radicals generated by the effect of the nanoparticles on the bacteria was calculated by subtracting the absorbance obtained for the negative controls from that of the test samples.

### 2.6. Crystal Violet Assay to Determine Biofilm-Forming Ability

The effect of ZnONPs on the ability of *B. cereus* to form a biofilm was tested by spectrophotometry using crystal violet [40] in the wells of Bioscreen C microplates. Cultures of *B. cereus*—control and with ZnONPs—were cultured for 48 h, after which the supernatant was poured off. The wells were washed with saline solution, dried, and stained with 400 µL of 0.1% crystal violet for 20 min. The stain was bound by the biofilm in the wells. Then the wells were washed and dried, and the stain bound to the biofilm was dissolved in 400 μL of 30% acetic acid. The absorbance of the crystal violet solution was measured by spectrophotometry at 600 nm [41]. The results were presented as % absorbance of the control sample.

### 2.7. Ability to Decompose (Decolourize) Evans Blue Dye

The ability to decompose azo dyes in the presence of ZnONPs was tested in a test tube test. The dye used in the test was Evans Blue (EB)—C_34_H_24_N_6_Na_4_O_14_S_4_, molar mass 960.81 g/mol. A preliminary experiment was conducted in which the OD of the bacterial culture on nutrient broth supplemented with various concentrations of Evans Blue was measured after 48 h of growth. The azo dye at a concentration of 0.1 mg/mL did not inhibit bacterial growth. In addition, growth medium with 0.1 mg/mL Evans Blue and various concentrations of ZnONPs, without bacteria, was incubated for 48 h, which confirmed that incubation of the dye with nanoparticles does not change the intensity of the colour of the solution, indicating that the dye is stable in these conditions. 

A solution of Evans Blue at a concentration of 0.1 mg/mL was added to the bacterial culture. After 48 h incubation the cultures were centrifuged and the absorbance of the supernatant was measured at λ = 606 nm. Growth medium with 0.1 mg/mL Evans Blue, without bacteria, was used to determine the baseline absorbance A_0_. The degree of decolouration was determined from the following formula:

Decolouration (%) = [(A_0_ − A_1_)/A_0_] × 100

A_0_ is the absorbance of control sample of dye, A_1_ is the absorbance of the sample following incubation with bacteria [42].

### 2.8. Evaluation of the Synergistic Effect of ZnO Nanoparticles and Selected Phenolic Compounds on B. cereus by the Well Method

A suspension of *B. cereus* with a density of 1 × 10^5^ CFU/mL was plated in the amount of 0.5 mL on solid nutrient agar. Solutions of phenolic compounds in the amount of 50 μL were added to wells punched in plates. After 24 h of incubation, the *B. cereus* growth inhibition zone around the wells was measured. For the control samples, 0.3 mg of tetracycline (Sigma Aldrich) in the form of a solution was placed in the wells, and the following phenolic compounds were applied to the wells at a concentration of 0.5 mg: 4-hydroksybenzoic acid, sodium salicylate, trans-cinnamic acid, quercetin, gallic acid, coumarin, and p-coumaric acid (all from Sigma Aldrich). To test the effect of simultaneous application of nanoparticles and the substances named above, solutions containing a phenolic compound and ZnONPs at concentrations of 0.2 mg, 0.4 mg, 0.6 mg, and 0.8 mg were added to the wells. The antibacterial properties of the mixture of these substances were expressed by the size of the growth inhibition zone around the well.

All tests were performed in triplicate.

## 3. Results

### 3.1. Measure of Growth Parameters of B. cereus following Incubation with ZnONPs

Commercially produced ZnONPs were used in the experiment. They were obtained by hydrolysis of a zinc salt in a polyol medium heated to 160 °C (information from the manufacturer, Sigma Aldrich). They have a particle size of <100 nm measured by dynamic light scatting (DLS) and an average particle size of <35 nm measured using an aerodynamic particle sizer (APS) spectrometer, an average number weighted particle size of 67 ± 2 nm, and zeta potential of +46.1 ± 1.5 mV [36]. Our study confirmed that zinc oxide nanoparticles (ZnONPs) have biocidal properties against *B. cereus*. The MIC value for engineering ZnONPs against *B. cereus* was 1.6 mg/mL, and the MBC was 1.8 mg/mL. We tested whether nanoparticle concentrations below the MIC, i.e., 0.2, 0.4, 0.6, and 0.8 mg/mL ZnONPs, affect the growth parameters and metabolic activity of these bacteria. Table 1 shows that sublethal concentrations of ZnONPs inhibited planktonic growth of *B. cereus*. Even the lowest concentration of 0.2 mg/mL ZnONPs inhibited growth by about 14%, while 0.8 mg/mL, i.e., 50% MIC, inhibited growth by about 42%. The harmful impact of ZnONPs was also manifested as stimulation of the sporulation process, which leads to transformation of vegetative cells into spores resistant to the effects of harmful factors. After a 48 h culture of these bacteria in medium with no added substances (control), the level of sporulating cells was about 15%. Their number increased in the presence of ZnONPs, although it should be noted that the differences were not statistically significant. At 0.8 mg/mL ZnONPs, more than 20% of the entire population formed spores, which indicates that nanoparticles are capable of initiating this complex developmental process.

### 3.2. Oxidative Stress Measured by the NBT Assay

The NBT test was used to determine whether zinc oxide nanoparticles induce oxidative stress in *B. cereus* cells. Superoxide radicals are produced even in optimal growth conditions, i.e., in bacterial cells that are not exposed to ZnNPs. In the control cultures the absorbance of formazan was 0.127. Production of superoxide anion radical increases with increasing concentrations of zinc oxide nanoparticles. A positive correlation (*p* = 0.967) was found between the absorbance of formazan and the concentration of nanoparticles. The highest concentration of 0.8 mg/mL ZnONPs caused the most severe oxidative stress, with formazan absorbance equal to about 227% of the control value.

### 3.3. Biofilm Formation

Another manifestation of the response of bacteria to deterioration of growth conditions is biofilm formation. The process of biofilm formation by *B. cereus* in the presence of zinc oxide nanoparticles was tested by the crystal violet assay. The concentration of crystal violet released from the stained biofilm is proportional to the amount of biofilm formed [40]. The amount of biofilm was lowest in the control; the OD value for the crystal violet removed from the biofilm was 0.324. The amount of biofilm produced by the bacteria increased with the concentration of ZnNPs, as indicated by the increase in the absorbance of these solutions. The high correlation (*p* = 0.950) between the ZnONP concentration and the absorbance of the crystal violet solution indicates that nanoparticles stimulate biofilm formation. At a concentration of 0.8 mg/mL, the cells produced 80% more biofilm than in the control.

### 3.4. Evans Blue Decolouration

*B. cereus* bacteria are known for their capacity for biotransformation of various compounds, which are often environmental toxins. These include Evans Blue, an azo dye widely used in industry, whose decomposition products are more toxic than the dye itself [43,44]. The result of the pilot experiment indicates that Evans Blue at a concentration of 0.1 mg/mL does not inhibit the growth of *B. cereus* in liquid medium. These bacteria were capable of biotransformation of this azo dye; a 42% decolouration of Evans Blue was observed in the control sample (without zinc oxide nanoparticles). The negative correlation coefficient indicates that as the concentration of ZnONPs increases, the rate of decomposition of the dye decreases. Even at the lowest concentration of 0.2 mg/mL ZnONPs, there was a decrease in decolouration of Evans Blue, and at 0.8 mg/mL, about 76.5% of the initial concentration of undecomposed dye remained. 

### 3.5. Combined Activity of ZnONPs and Phenolic Compounds against B. cereus

The agar well diffusion method is often used to assess the antibacterial properties of various substances. In comparison with the agar disc diffusion method, in which about 10–20 μL of substance is dropped onto a paper disc, larger volumes of substances can be used in the well method [45]. In addition, the well method eliminates the need for cellulose paper as a carrier for nanoparticles, which can impede the release of nanoparticles into the agar. 

As shown in Table 2, growth inhibition zones of *B. cereus* appeared following the use of ZnONPs at concentrations below the MIC. ZnONPs at the concentrations applied had a much smaller biocidal effect than tetracycline. The growth inhibition zone of *B. cereus* at a concentration of 0.8 mg ZnONPs was 63% lower than for the antibiotic. Simultaneous application of ZnONPs and tetracycline did not increase the growth inhibition zones in comparison to the values obtained for the antibiotic alone. All phenolic compounds were used at a concentration of 0.5 mg/well, and no growth inhibition zones of *B. cereus* were observed for any of them. The growth inhibition zones were larger when the phenolic compound and ZnONPs at a concentration of 0.2 or 04 mg were applied together than in the case of nanoparticles alone. The growth inhibition zone increased by 50% when the lowest concentration of 0.2 mg ZnONPs was applied together with trans-cinnamic acid, and by 87% when applied together with coumaric acid or gallic acid. For all tested phenolic acids, the largest growth inhibition zones were observed when the compounds were used together with 0.4 mg ZnONPs. In these samples, the growth inhibition zones were 60–90% larger than in the case of nanoparticles alone. In the case of simultaneous application of phenolic compounds and higher concentrations of nanoparticles (0.6 mg and 0.8 mg), the growth inhibition effect was similar to that of nanoparticles alone. 

## 4. Discussion

*Bacillus cereus* is widespread in nature and can be found in raw materials used in the production of food and cosmetics, such as plant extracts and infusions [2]. As an opportunistic pathogen, *B. cereus* can pose a threat to human health. Nanomaterials including zinc oxide are used in many products that come into direct contact with the human body, such as household chemicals, bandages, packaging, or cosmetics. They have potential antibacterial effects on harmful microorganisms. Therefore, in the present study we investigated how *B. cereus* reacts to concentrations of ZnO nanoparticles below the MIC value. In the first step of this study, a biocidal effect against *B. cereus* was obtained for commercially produced ZnONPs at MIC = 1.6 mg/mL and MBC = 1.8 mg/mL. The literature reports varied MIC values against bacteria for zinc oxide nanoparticles. MIC and MBC values much lower than in the present study were obtained for related species of the genus *Bacillus* [46,47]. Zinc oxide nanoparticles can have biocidal effects against bacteria posing a threat to human health. They usually exhibit stronger activity against Gram-negative bacteria than against Gram-positive bacteria [40]. MIC values of 1.25–10 mg/mL were obtained for Gram-negative bacteria of the family *Enterobacteriaceae*, and 2.5–5 mg/mL for Gram-positive *Staphylococcus* sp. [48]. ZnO nanoparticles obtained using biological methods usually exhibit strong antibacterial activity against *Escherichia coli*, *Staphylococcus aureus*, *Pseudomonas aeruginosa*, and *Bacillus subtilis* [49,50]. The reactivity and biocidal properties of zinc oxide nanoparticles are influenced by many factors: their shape, size, surface charge, and concentration, and the presence of additional chemical substances in the reaction environment. The same concentration (1 mg/mL) of ZnONPs can induce varying degrees of biocidal effects against *B. subtilis* depending on environmental conditions [45]. The discrepant MIC and MBC values presented for ZnONPs indicate the need for thorough testing in the case of application of zinc oxide nanoparticles as an effective antibacterial agent. 

*Bacillus cereus* is generally resistant to penicillin, ampicillin, and cephalosporin because it produces beta-lactamase and cephalosporinase. Most strains are susceptible to erythromycin, chloramphenicol, vancomycin, ciprofloxacin, and tetracycline [51]. Nanoparticles of metals, especially silver and copper, can enhance the antibacterial activity of selected antibiotics, which may have applications in the treatment of bacterial infections [52,53]. ZnONPs also increase the antibacterial effect of ciprofloxacin against *S. aureus* and *E. coli* [54]. In our experiment, the strongest biocidal effect was exerted by tetracycline. Moreover, the biocidal effect of tetracycline was the same whether it was applied alone or in combination with sublethal concentrations of ZnONPs (Table 2). According to the literature, however, zinc oxide nanoparticles can have synergistic or additive effects, depending on which antibiotic they are combined with [55]. 

Phenolic compounds, apart from antioxidant activity, can exhibit significant antibacterial activity. They are natural components of plants, and as plant extracts, they are used as antioxidants and preservatives in food products and cosmetics [56]. In the present study, phenolic compounds were used at a concentration of 0.5 mg/well and did not affect the growth of *B. cereus*. According to literature reports, however, phenolic compounds may stimulate cell growth or exert an antibacterial effect, depending on both the bacterial strains and the concentration and chemical structure of the phenolic compound [57]. Bacilli are also known to be capable of decomposing numerous complex organic compounds, including phenols [58,59].

In the present study, when phenolic compounds were used together with ZnONPs with a concentration of 0.2 or 0.4 mg/well, the growth inhibition zones increased in comparison to the effect of nanoparticles alone (Table 2). The mechanism of this increased antimicrobial effect is not obvious, and further research is needed to explain it. 

Phenolic compounds interact with components of the bacterial cell wall, increase the permeability of plasma membranes, and cause ion efflux from the bacterial cell [60], which may facilitate penetration into the cell by nanoparticles. The enhanced antimicrobial effect of zinc oxide nanoparticles used together with phenolic compounds (Table 2) may also be due to increased oxidative stress. Phenolic compounds can enter into redox reactions with metal nanoparticles and increase free radical generation [61]. Literature data confirm that the presence of additional chemical substances influences the biocidal properties of zinc oxide nanoparticles. When water from two different rivers and the same concentrations of ZnONPs were used, the biocidal effect was varied [62]. Metal nanoparticles synthesized using a plant extract have also been shown to exhibit greater biocidal activity than nanoparticles prepared chemically [63]. ZnO nanoparticles alone induce generation of reactive oxygen species (ROS) and oxidative stress. They cause lipid peroxidation, oxidative modifications of proteins, organelle dysfunction, inflammation, and DNA damage [64].

The experiments confirmed that even sublethal concentrations of zinc oxide nanoparticles reduce planktonic cell growth, which was determined by measuring OD, and increase the defensive response of the bacteria, which was expressed as transformation of vegetative cells into spores and biofilm formation (Table 1). Bacteria of the genus *Bacillus* form spores during prolonged incubation in growth medium or in conditions of limited nutrients. Spores are resistant to starvation conditions, drying out, and high temperature; they survive dry storage and cooking [8]. Our experiment showed that sublethal concentrations of zinc oxide nanoparticles increase the number of spores in a culture of *B. cereus* (Table 1). This observation may be significant for the use of ZnONPs in food packaging, in which various forms of zinc oxide nanoparticles are used in concentrations from 0.5% to 5%, or less often 20% [65]. 

Both biofilm formation and spore formation are strategies that help bacteria to survive when harmful substances appear in the environment. A biofilm is formed in natural conditions, e.g., on the roots of plants, and performs important functions in bacteria–rhizosphere interactions [66]. A biofilm formed on body tissues such as the urinary tract or on medical implants makes it more difficult to treat bacterial infections, because cells in the form of a biofilm are more resistant to antibiotics and can spread throughout the body [67]. Nanotechnology provides effective agents protecting against the formation of bacterial biofilms or removing them. A decrease in biofilm formation by *Pseudomonas aeruginosa*, *Vibrio cholera*, and *Brevibacterium* has been described following application of gold nanoparticles [68]. Silver nanoparticles were able to inhibit biofilm formation by *Staphylococcus aureus* [69]. Many publications postulate the use of ZnONPs as an antibacterial and anti-biofilm agent [70]. ZnO is considered a safe material by the American Food and Drug Administration (21 CFR 182.8991) [71]. Zinc oxide nanoparticles can limit the formation of bacterial biofilm because they have biocidal and biostatic effects and decrease the hydrophobicity index [72]. 

ZnO nanoparticles have also been reported to remove biofilms formed by *Streptococcus pneumoniae* [73] or *Pseudomonas aeruginosa* [74]. Our experiment showed that concentrations of ZnONPs below the MIC50 can stimulate biofilm formation by *B. cereus*. The increase observed in the amount of biofilm as an effect of sublethal concentrations of ZnONPs may be due to the specific features of the nanoparticles used, such as their size (100 nm), the chemical method of obtaining them, and the sublethal concentrations used. An anti-biofilm effect is described in the literature at concentrations of nanoparticles equal to the MIC, while in our experiment biofilm formation increased at concentrations below MIC50.

Sublethal concentrations of ZnONPs cause oxidative stress in *B. cereus*, which we confirmed in the NBT assay (Table 1). Free radical generation by ZnONPs is one of the most commonly described mechanisms of their antibacterial effect [75]. The direct interaction of nanoparticles with the bacterial cell envelope causes accumulation of ZnO nanoparticles on the cell surface and their adsorption in the peptidoglycan layer. Chemical compounds present on the cell surface of bacteria (carboxyl, phosphate, and amine groups) give it a negative charge, which is conducive to ROS generation [76]. The presence of oxygen in the environment is conducive to the formation of superoxide anion radical (O_2_^−^), which in turn can generate additional ROS [75]. The cytotoxic effect of ZnONPs has been shown to be associated with the production of superoxide anion radical, which causes oxidative stress [75]. In the present study, the presence of ZnONPs at concentrations below the MIC in a culture of *B. cereus* increased production of superoxide anion radical (Table 1). 

Similar observations have been made for *B. subtilis*, with metal nanoparticles inducing a significant increase in ROS concentrations and toxic effects associated with oxidative stress [77]. Another important observation arising from our experiments is that zinc oxide nanoparticles influence metabolic processes in *Bacillus* which are important for the biotransformation of various compounds. *B. cereus* is capable of decomposing anthropogenic pollutants, including azo dyes, nitriles, and pesticides such as beta-cypermethrin and pentachlorophenol [78,79,80]. It also plays an important role in the environment by breaking down natural complex chemical compounds such as resveratrol or polyphenolic compounds [58,59]. In our study, ZnONPs were shown to have a negative effect on decomposition of the azo dye Evans Blue. The growth rate was the same in the control culture as in the culture with Evans Blue at a concentration of 0.1 mg/mL. The concentration of the dye was relatively low compared with those applied in other studies, because at high concentrations the dye may inhibit the growth of bacterial cells [81]. In our study, in the control culture containing Evans Blue, a 42.67% decolouration took place within 48 h. The rate of decolouration of the dye decreased as the concentration of zinc oxide nanoparticles increased. *Bacillus* species, such as *Pseudomonas*, *Escherichia*, *Citrobacter*, *Shewanella*, *Desulfovibrio*, *Proteus*, *Sphingomonas*, *Aeromonas*, and *Alcaligenes*, are capable of biotransformation of azo dyes [81,82]. High efficiency of decolouration of azo dyes at high concentrations has been obtained for isolates of *Bacillus cereus*, *B. vallismortis*, *B. pumilus*, *B. subtilis*, and *B. megaterium* [83,84]. *Bacillus* spp. cells remove azo dyes from the environment by decomposing them, not by absorbing them into the cell [81]. Zinc oxide nanoparticles have photocatalytic properties, and therefore can be used together with UV radiation to increase the decomposition rate of azo dyes [85]; however, a UV lamp was not used in our study, and the cultures were incubated in an incubator (in the dark). Inhibition of decolouration suggests that in *B. cereus* cells metabolic processes leading to biotransformation of Evans Blue were inhibited by ZnONPs. 

## 5. Conclusions

The common use of nanoparticles in industry may lead to their uncontrolled presence in the environment. Many literature reports emphasize the bactericidal and biostatic effects of metal nanoparticles, but knowledge of their sublethal effects on bacteria is limited. Like other bacteria, *B. cereus* plays important roles in the environment, but is also an opportunistic pathogen. Understanding its reactions to sublethal concentrations of zinc oxide nanoparticles is important in the context of protection of health and the natural resources of the soil environment. An unfavourable aspect of the effects of sublethal concentrations of nanoparticles on *B. cereus* as an opportunistic pathogen is undoubtedly the stimulation of biofilm formation and the formation of endospores resistant to the effects of physicochemical factors. On the other hand, the potentially beneficial effects of sublethal concentrations of zinc oxide nanoparticles for controlling these bacteria are the limitation of planktonic growth and intensification of oxidative stress, which is one of the main biocidal mechanisms postulated for nanoparticles. The antimicrobial activity of sublethal concentrations of ZnONP for *B. cereus* increases in the presence of phenolic compounds. This phenomenon may be exploited in developing new formulations for cosmetics. It should also be noted that for *B. cereus*, as rhizosphere bacteria supporting plant growth and taking part in the biodegradation of various compounds, including xenobiotics, the presence of sublethal concentrations of zinc oxide nanoparticles may pose a threat, leading to metabolic changes and ultimately to the inhibition of the growth of these bacteria. 

## Figures and Tables

**Table 1:** Effect of ZnONPs on growth and specific metabolic activity of *Bacillus cereus*.

ZnONPs [mg/mL]	Optical Density [Absorbance]	Spore Formation [%]	NBT Reduction [Formazan Absorbance]	Biofilm Formation [Crystal Violet Absorbance]	Evans Blue Decolouration [% of Decolouration]
0 (control)	1.447 ^a^	15 ^a^	0.127 ^a^	0.3245 ^a^	42.67 ^a^
0.2	1.259 ^b^	16 ^a^	0.155 ^ab^	0.394 ^b^	36.43 ^a^
0.4	1.112 ^c^	20 ^a^	0.244 ^b^	0.428 ^c^	33.91 ^a^
0.6	0.930 ^d^	20 ^a^	0.260 ^bc^	0.448 ^c^	22.88 ^b^
0.8	0.839 ^e^	22.5 ^a^	0.289 ^cd^	0.586 ^d^	23.50 ^b^
Pearson correlation coefficients	−0.994	0.965	0.967	0.950	−0.959

Values sharing the same letter in column are not significantly different.

**Table 2:** Antimicrobial potential of ZnONPs and selected phenolic compounds against *Bacillus cereus*.

Analysed Substances	Antimicrobial Activity of Chemical Compounds [mm Inhibition Zone]					Pearson Correlation Coefficients
ZnONPs	Control	0.2 mg	0.4 mg	0.6 mg	0.8 mg	
	0	8	10	12	13	0.902
	Chemical compound alone	0.2 mg ZnONPs + S	0.4 mg ZnONPs + S	0.6 mg ZnONPs + S	0.8 mg ZnONPs + S	
Tetracycline	36	36	36	35	35	−0.866
4-Hydroksybenzoic acid	0	13	16	13	13	0.654
Sodium salicylate	0	13	17	14	13	0.649
Trans-cinnamic acid	0	12	16	15	13	0.822
Quercetin	0	13	17	13	12	0.589
Gallic acid	0	15	16	10	10	0.374
Coumarin	0	13	16	13	11	0.562
Coumaric acid	0	15	19	16	15	0.693

## Data Availability

The data presented in this study are available on request from the corresponding author.
